# Risk Factors for Falls in Hospital In-Patients: A Prospective Nested Case Control Study

**DOI:** 10.15171/ijhpm.2019.11

**Published:** 2019-03-09

**Authors:** Zhila Najafpour, Zahra Godarzi, Mohammad Arab, Mehdi Yaseri

**Affiliations:** ^1^Department of Health Care Management, School of Public Health, Ahvaz Jundishapur University of Medical Sciences, Ahvaz, Iran.; ^2^Department of Pharmacoeconomics and Pharmaceutical Administration, Faculty of Pharmacy, Tehran University of Medical Sciences, Tehran, Iran.; ^3^School of Public Health, Tehran University of Medical Sciences, Tehran, Iran.; ^4^Department of Epidemiology and Biostatistics, School of Public Health, Tehran University of Medical Sciences, Tehran, Iran.

**Keywords:** Accidental Fall, Hospital, Risk Factors, Nested Case Control

## Abstract

**Background:** Patient falls are considered a challenge to the patient’s safety in hospitals, which, in addition to increasing the length of stay and costs, may also result in severe injuries or even the death of the patient. This study aims to investigate the associations between risk factors among fallers in comparison with the control group.

**Methods:** A prospective nested case control study was performed on 185 patients who fell and 1141 controls were matched with the patients at risk of fall in the same ward and during the same time. This study was conducted in a university educational hospital in Tehran with 800 beds during a 9-month period. The data included demographics, comorbidities, admission details, types of medication, clinical conditions, and activities before or during the fall. The data was collected from clinical records, hospital information system, error reporting system and observations, and the interviews with the fallers, their families and care givers (physicians, nurses, etc). Data analysis was conducted through time-based matching using a multi-level analysis.

**Results:** In a multilevel model including patient-related, medication, and care-related variables, the factors that were significantly associated with an increased risk of patient falls included: longer length of stay (odds ratio [OR] = 1.01; CI=0.32 to 0.73), using chemotherapy drugs, sedatives, anticonvulsants, benzodiazepines, and angiotensin-converting enzyme (ACE) inhibitors, visual acuity (OR=6.93; CI=4.22 to 11.38), balance condition (OR= 6.41; CI=4.51 to 9.11), manual transfer aid (OR=8.47; CI=5.65 to 12.69), urinary incontinence (OR= 8.47, CI= 5.65 to 12.69), and cancer (OR=2.86, CI=1.84-4.44). These factors were found to be associating with more odds for a falling accident among patients. Several characteristics such as fall history (OR=0.48; CI= 1.003 to 1.02), poly-pharmacy (OR=1.37, CI=00.85 to 2.2), stroke (OR=0.94, CI= 0.44 to 2.02), and nurse to patient ratio (incidence rate ratio=1.01, CI=0.01 to 0.03) were not significantly associated with falling in hospitals.

**Conclusion:** It seems that a combination of both patient-related factors and history of medication should be considered. Moreover, modifiable clinical characteristics of patients such as vision improvement, provision of manual transfer aid, diabetes control, regular toilet program, and drug modification should be considered during the formulation of interventions.

## Background

Patient falls within hospitals continue to be a serious concern and are the most common adverse events leading to injury, longer lengths of stay, and increased costs among hospitalized patients.^1,2^

The rate of falls varies considerably by hospital and by unit type ^3,4^ However, there is an agreement that falls are a common problem in hospitals: fall rates range from 2.6 to 7 per 1000 patient days, and almost 23% to 42% of the falls contribute to at least one type of injury and 2% to 9% of them lead to serious injuries.^5-7^ The consequences of fall-related injuries are also associated with substantially raised costs (fallers with serious injuries are charged $13 316 more than non-faller inpatients and, on average, have a 6-8 day longer stay at hospitals).^8,9^ In addition to physical injuries, patients are disposed to mental harms such as anxiety, fear of falling, and loss of self-confidence.^10^

The first step in preventing falls is the identification of high-risk patients. In this regard, risk factors are considered as the key to identifying patients susceptible to falling and selecting effective measures in fall prevention. A plethora of research in the literature has identified a number of risk factors as influential in patients’ falls.^11^

Different studies have marked various explications of risk factors,^11^ and there is no definitive consensus on the type and number of risk factors leading to a fall. It is imperative that comprehensive information be gathered about the risk factors of falling among hospitalized patients for the purpose of designing preventive interventions. This is mainly due to the fact that despite the implementation of various trials worldwide, no fully effective proceeding has yet been developed to targeted fall preventive interventions.^12^

Risk factors are classified into two types of (*a*) intrinsic factors including age, gender, musculoskeletal disorders, patient’s imbalance and using drugs; and (*b*) extrinsic factors including the weakness of the health system in the maintenance and design of medical equipment, human resources, communication, training, and team work. The negative interactions between intrinsic and extrinsic risk factors lead to serious physical injuries.^13^

Due to the risk of significant injury, increased costs, and emotional harms, reducing the number of falls in hospitals is a major priority. Despite concerted efforts to prevent patient falls in hospitals, still there are identifiable gaps in the literature and hospitals continue to struggle with the consequences of patient falls. Fall prevention programs are most efficient when linked to the modifiable risk factors. Therefore, it is imperative to develop some methods to accurately identify those at the highest risk in order to formulate targeted interventions. Further research is required to confirm the risk factors. Along this line, we conducted a nested case-control study to determine patient-related, medication, and care-related predictors of inpatient falls and to investigate the associations between risk factors and falling incidents in a general hospital.

## Methods

The present study is a prospective nested case-control investigation conducted on patients admitted in a general hospital with an annual admission of 24 000 patients during a period of 9 months (June 2016–March 2017). The units were general, heart surgery, neurosurgery, orthopedics, general surgery, hematology, emergency, and gynecology. A total of 1326 patients were enrolled in the study. Of these, 185 cases and 1141 controls were recruited into the study.

### Case and Control Groups

In our study, the cases were patients admitted during the study period who had a falling incident at the hospital. In this study, a patient fall is defined “as an unplanned descent to the floor with or without injury to the patient.”^14^ A total of 185 falls were recorded during the study period in different wards. Meanwhile, we analyzed only the first falls, excluding the second falls to reduce the bias.^15^

For a comparison group, all patients admitted to the participating wards were assessed by nurses (at least within 24 hours of admission) using a Morse Fall Scale and the patients were considered as high-risk and eligible for inclusion if they had scores higher than 45. A total of 1141 patients were enrolled in the study as the control group (high-risk patient). All cases were matched with high-risk patients hospitalized in the same ward and at the same time.

### Data Collection

A data collection tool developed by the research team was employed for recording variables from fallers and controls. These variables are presented in Table 1. The data were collected by the researchers from patient records, hospital information system, error reporting system, environmental observations, and interviews with the cases and their families, physicians, and nurses (a median of 1 day after occurring a fall to facilitate collecting information before the patients were discharged) (See Supplementary file 1).

**Table 1:** Variables on Fall Data Collection Tool

**Variables Collected for Both Fallers and Controls**
Patient demographics
Medications taken within 24 hours
Fall risk level (assigned via Morse Falls Scale by the nurse at admission)
History of fall
Health status variables^a^
**Additional Variables Collected Only for Patients Who Fell**
Place, date, and time of fall
Activity level at time of fall
Location of fall
Type and severity of injury^b^

^a^ History or presence of depressive disorder, visual impairment, motor disorders, manual transfer aid, history of stroke, Parkinson’s disease, diabetes, urinary incontinence, and cancer. ^b^ Type and severity of injury such as minor injury (patients’ complaints of pain, the need for ice packs, bandages, cleaning wounds, swelling, analgesics, etc), moderate injury (cramps, stretching of the muscles, requiring stitches or bed rest), severe injury or death (fracture, surgery, etc).^16^

The post-fall conditions of the patient were recorded by the physician. After any patient fell, physicians examined the details of the injuries and prescribed treatment at a maximum interval of 1 to 2 days.

A wealth of studies has called for the need for employing multiple methods for collecting valid data on patients’ falls.^17,18^ Therefore, in this study, in addition to hospital error reporting systems, all the patients admitted during the study period were interviewed before being discharged from the hospital on their falling experiences. Their records were also reviewed on a daily basis with the aim of detecting the error reporting gaps.

Identification of high-risk patients was conducted by the Morse Fall Scale in all the wards. The Morse Fall Scale is a fall risk assessment tool that has been developed using a rigorous research design^19^ and is valid for identifying the patients at high risk of falling.^20^ It consists of 6 items: history of falling (3 months ago) (possible score: 0 or 25), presence of secondary diagnosis (0 or 15), use of an ambulation aid (0, 15, or 30), IV or IV access (0 or 20), type of gait (0, 10, 20) and mental status (0 or 15). The total score can range from 0-125^20^ and we considered only patients with scores of more than 45 as high-risk patients.

### Medication

The patients’ medications were identified on admission and discharge. The details of medications received by both cases and controls were obtained through the hospital information system, patient records, and interviewing the patients or their families about their medication history during the last two weeks. All drugs used were evaluated and categorized by a clinical pharmacologist. Medications were classified into 21 groups: sedatives, antiarrhythmic agents, anti-diabetic agents, anticoagulants, vasodilators, anticonvulsants, benzodiazepines, angiotensin-converting enzyme (ACE) inhibitors, beta blockers, narcotic analgesics, antipsychotics, calcium channel blockers, alpha blockers, chemotherapy, non-steroidal, hypertensive agents, anti-infective agents, antihistamine drugs, autonomic drugs, gastrointestinal drugs, and respiratory tract agents.

### Data Analysis

Data were entered into Microsoft Excel, cleaned, and then transferred to STATA version 11.0 (StataCorp Lp). The values were expressed as means, standard deviations, ranges, and precents. Independent variables were age, gender, Morse scale score, history of falls, type and number of medications, length of stay, and comorbidities. The dependent variable was the rate of fall. To consider the matching in our study, we used a multilevel analysis (patient and ward levels). The precision of the estimates was presented by 95% CI and a *P* value less than .05 was considered statistically significant. Chi-square and Fisher exact tests were used to compare characteristics of the patients (case and control groups) for categorical variables. To compare continuous variables, analysis of variance and the Kruskal-Wallis test were used as appropriate tests. The magnitude of the relationship between risk factors and falling was quantified using the odds ratio (OR). Logistic regression was used to calculate both the crude OR and the adjusted OR. We adjusted the model for age, Morse scale score, history of prior falls and length of stay. Also, a Poisson regression was used to assess the effect of ward variable and patient fall (such as the ratio of nurses to patients).

## Results

### Characteristics of Cases and Controls

In this study, 185 patients were in the case group and 1141 patients comprised the control group. The mean ages of the patients in case and control groups were 52.9 and 58.6, respectively. About 57% of the patients in the case group and 45% of the patients in the control group were females. The correlation results among the studied variables revealed no statistically significant difference in terms of gender and age. However, there was a significant difference between the risk factors such as prior fall history (3 months ago) and length of stay (*P* ≤ .05). However, only odds of falling with longer length of stay were increased (Table 2).

**Table 2:** Characteristics of Cases and Controls

**Variables**	**Control** ^a^ ** (n = 1141)**	**Case** ^a^ ** (n = 185)**	**OR (Crude)** ^a^ ** (95% CI)**	***P*** ** Value**
Age (y), mean, SD (range)	58.6, 17.2 (57.6-59.6)	52.9, 18.5 (50.2-55.6)	0.99 (0.98 to 1.00)	.069
Gender				
Male, No. (%)	630.0 (55%)	106.0 (57)	0.98 (0.72 to 1.34)	.901
Female, No. (%)	511.0 (45%)	79.0 (43)		
Mean 'Morse Falls Scale Score', mean, SD (range)	60.4, 15.8 (59.4-61.3)	47.4, 19.0 (44.6-50.1)	0.93 (0.93 to 0.95)	<.001
Fall history (3 mounts ago), No. (%)	171.0 (14%)	42.0 (22.7%)	0.48 (0.32 to 0.73)	<.001
Mean length of stay, days, mean, SD (range)	10.2, 12.8 (7.2-8. 4)	13.84, 14.3 (11.8-15.9)	1.01 (1.00 to 1.02)	<.001

Abbreviations: OR, odds ratio; SD, standard deviation. ^a^The estimates accompanied with 95% CI in the parenthesis‏ based on multilevel logistic regression.

### Medication

Table 3 shows the OR for taking drugs and falling. Findings of our study confirmed that using sedative, anticonvulsants, anti-diabetic agents, benzodiazepines, ACE inhibitors, anti-infective agents, antihistamine, and chemotherapy drugs were significantly different between the case and the control groups. The use of the mentioned drugs was associated with higher odds for a falling accident among the patients. However, there were not any strong associations between the crude and the adjusted data for poly-pharmacy (use of ≤ 5 medicines) and patient fall. Poly-pharmacy was used in 159 cases (85.9%) and 918 controls (80.6%) (Table 3).

**Table 3:** Medications Administered to Cases and Controls

**Pharmaceutical Use**	**Case** **No. (%)**	**Control** ** No. (%)**	**OR (Crude) (95% CI)**	***P *** **Value**
Sedative/hypnotics	131 (70.8)	709 (62.2)	1.47 (1.04 to 2.06)	.02
Antiarrhythmic agents	62 (33.5)	384 (33.7)	0.99 (0.71 to 1.37)	.95
Anti-diabetic agents	19 (10.3)	32 (2.8)	0.16 (0.13 to 0.18)	<.001
Anticoagulants	128 (69.2)	815 (71.6)	0.89 (0.63 to 1.25)	.51
Vasodilators/vasoactive	37 (20.0)	275 (24.1)	0.78 (0.53 to 1.15)	.219
Anticonvulsants, miscellaneous	108 (58.4)	576 (50.6)	1.37 (1.00 to 1.87)	.04
Antidepressants	18 (9.7)	69 (6.1)	1.67 (0.97 to 2.87)	.06
Benzodiazepines	96 (51.9)	498 (43.7)	1.38 (1.01 to 1.89)	.03
ACE inhibitors	96 (51.9)	714 (62.7)	0.64 (0.46 to 0.87)	.005
Beta blockers	91 (49.2)	602 (52.9)	0.86 (0.63 to 1.17)	.35
Narcotic analgesics	143 (77.3)	874 (74.7)	1.03 (0.71 to 1.49)	.86
Antipsychotics	49 (26.5)	345 (30.2)	1.36 (0.84 to 2.21)	.19
Calcium channel blockers	89 (48.1)	606 (53.2)	0.18 (0.59 to 1.11)	.19
Alpha blockers	90 (48.6)	38 (2.9)	0.82 (0.60 to 1.12)	.22
Chemotherapy	23 (12.0)	67 (5.9)	2.27 (1.37 to 3.75)	.001
Non-steroidal/anti-inflammatory	140 (75.7)	874 (76.7)	1.07 (1.06 to 5.8)	.70
Hypertensive agents	50 (27.0)	368 (32.3)	0.77 (0.54 to 1.09)	.15
Anti-infective agents	148 (80.0)	789 (69.3)	1.70 (1.21 to 2.59)	.003
Antihistamine drugs	23 (12.4)	78 (6.8)	1.93 (1.17 to 3.16)	.009
Autonomic drugs	53 (28.6)	331 (29.1)	0.98 (0.69 to 1.38)	.90
Gastrointestinal drugs	152 (82.2)	910 (79.9)	1.15 (0.77 to 1.73)	.40
Respiratory tract agents	38 (20.5)	238 (20.9)	0.97 (0.66 to 1.43)	.90
Ploy-pharmacy	159 (85.0)	918 (80.0)	1.47 (0.94 to 2.28)	.08
Ploy-pharmacy (adjusted)^a^	159 (85.0)	918 (80.0)	1.37 (0.85 to 2.20)	.18

Abbreviations: OR, odds ratio; ACE, angiotensin-converting enzyme. Based on multilevel logistic regression. ^a^Adjusted with variables of age, gender, Morse scale score, history of prior fall, and length of stay.

### Clinical Conditions of Patients

In terms of the clinical conditions of patients under study, the cases were reported to have significantly higher rates of depression, visual impairment, balance disorder, manual transfer aid, urinary incontinence, cancer, Parkinson’s disease, and diabetes mellitus. The results showed that patients with this comorbidity had higher odds for falling accidents than others (Table 4).

**Table 4:** Patient Care-Related Factors

**Variable**	**Case** **No. (%)**	**Control** **No. (%)**	** Adjusted OR** ^a^	**P>|z|**	**95% CI**	
					Lower	Upper
Depression	27 (14.6)	32 (2.8)	5.90	<0.001	3.44	10.12
Vision problem	38 (20.5)	42 (3.7)	6.93	<0.001	4.22	11.38
Gait disorder	87 (47.0)	140 (12.3)	6.41	<0.001	4.51	9.11
Use of walking aids	71 (40.5)	91 (8.1)	8.47	<0.001	5.65	12.69
Stroke	8 (4.3)	52 (14.6)	0.94	0.884	0.44	2.02
Incontinence	28 (15.1)	40 (3.5)	4.86	<0.001	2.91	8.10
Parkinson disease	3 (1.6)	3 (0.3)	4.96	<0.001	0.70	6.84
Cancer	33 (17.8)	80 (7.0)	2.86	<0.001	1.84	4.44
Diabetic	19 (10.3)	32 (2.8)	2.88	<0.001	1.54	5.38

Abbreviation: OR, odds ratio. ^a^Based on multilevel logistic regression and adjusted with variables of age, gender, Morse scale score, history of prior fall, and length of stay.

### Fall Reports

Among fall incidents, 63% of the fallers experienced varying degrees of injuries from minor to major (minor: 46%, moderate: 12%, and sever injury or death: 4%). Some 67% of the fallers were unassisted and had no observer or a manual transfer aid. Meanwhile, activity restriction (complete bed rest) had been prescribed by physicians in 24% of fallers before the fall occurred. The most frequent place before or at the time of fall was the bathroom (39%), and the majority of falls (41%) occurred during the night shift. Although the nurse-to-patient ratio was lower in night shifts (incidence rate ratio  = 1.01, CI = 0.01 to 0.03, *P* > .05), it was not significantly associated with increased risk of falls. From the total falls, 51% of the actual fall incidents were reported by nurses and the reporting error system. Others were disclosed through interviews before patients’ discharge (Table 5).

**Table 5:** Activities of Patients Prior to or During Falls

**Activity**	**No.**	**Percent**
Getting up from bed, wheelchair, chair	48	26
Walking	62	34
Being transported	2	1
Bathroom (toileting, shower)	73	39
Sum	185	100

## Discussion

Hospital falls have a multiple etiology. The results of this study showed that patient-related factors such as longer length of stay, and clinical risk factors such as visual impairment, balance difficulties, manual transfer aid, and urinary incontinence as well as medication with drugs such as sedatives, anticonvulsants, anti-diabetic agents, benzodiazepines, ACE inhibitors, anti-infective agents, antihistamine, and chemotherapy drugs increased the risk of patient falls.

### Patient-Related Factors

The findings of this study indicated that the demographic variables (ie, age and gender) were not risk factors in our study. In 2009, the American Centre for Disease Control reported that the incidence of fall and fall-related injuries for males and the elderly above 85 years of age was four times greater than the patients aged 65-74.^21,22^ In the DUNLOP study, conditions associated with age such as arthritis, diabetes, heart disease, urinary incontinence, and visual impairment were suggested as predictors of the increased risk of falling among patients.^23^ In the study of Quigley et al,^24^ there has been a positive correlation between age increase and fall-induced injuries among hospitalized patients. However, in some studies, similar to ours, there was no correlation between age or gender and patient falls.^5,25-27^ Therefore, various studies have revealed inconsistent findings. However, aging, in combination with factors such as poor mobility or confusion, may result in a fall and the subsequent injuries.

The findings have indicated that the risk of repeated falls in patients with fall history is higher. Our results did not, however, confirm any association with the increased risk of patient falls. The results of other studies confirm that circumstances or characteristics of prior falls were repeated in subsequent falls.^27,28^ In addition, the history of falls may mask the influence of factors causing these earlier falls in a way that it can be considered as an indicator of an underlying problem like impaired balance.^29^ Deandrea reported this factor as helpful in identifying individuals at high risk of falling and recommended the inclusion of such factors in the fall risk assessment tools administered during the hospital admission.^27^ In our study, neglecting the prior fall history among cases during the initial assessment due to difficulties such as confusion, mental health problems, and the lack of cooperation between the patient and the nurses in addition to the nurses’ failure to ask about the history of falls led to the loss of this piece of information. In this regard, the role of nurses in effective communication with patients is paramount. It is suggested that in patients with one or more experiences of fall, the conditions of the previous falls and their underlying causes should be thoroughly investigated. In the same line, Healey et al has recommended locating the beds of such patients as close to the nursing station as possible.^30^

Balance difficulties and use of walking aids are important risk factors that have increased the rate of falls. In the studies by Quigley et al^31^ and Mayo et al,^28^ the decreased mobility and use of an assistive device were associated with injurious falls. However, the study of Baloh et al suggested that there was no clear causal relationship between the poorer balance and fall incidents.^32^ In addition to increasing the access to walkers and canes at the bedside, families should be encouraged to bring the patient’s walkers or assistive devices from home to be used in the hospital.^33^ Moreover, physical therapy can significantly improve the motor performance which is known as a risk factor for falling.^34^

Another risk factor associated with falls was the cancer disease already confirmed in the study of Capone et al^35^ and Spolesra et al,^36^ similar to the present study. Hitcho et al reported that patients under oncology treatment may be more prone to fall-related injuries due to anemia, thrombocytopenia, and risk for pathologic fracture.^33^ However, the risk factors among patients with cancer are different from other patients so that the most common reported factors such as pain, type of cancer, metastasis, antipsychotics, antidepressants, and blood transfusions are influential in a fall incident.^35^ It seems that there is a need to develop a specific assessment tool or to use complementary tools to predict more precisely the risk of fall among patients receiving oncology treatment.

Other relevant factors addressed in this study were visual impairments as well as incontinence and frequent urination. Patients with visual deficits are more likely to fall due to their reduced visual acuity, poor balance, false perception of environmental elements, and sensory loss.^37,38^ Leat et al suggested implementing an assessment of vision at hospital admissions would be useful for identifying patients who are at risk for falls due to poor vision,^39^ but it seems that these preventive interventions (vision assessment and eyeglass prescription) may be unrealistic for a hospital setting, hence we emphasize that the patients with a visual impairment use glasses while they are in the hospital, encourage regular eye examinations especially for the elderly, and keep adequate light levels at night in hospitals.

The effect of incontinence and frequent urination is confirmed in the study of Krauss et al.^40^ Also, Oliver et al suggest the provision of safe footwear and attention to the causes of incontinence urination. They illustrate that acutely ill patients are most mobile when walking between their bedsides to the toilet.^41^ In addition, creating a regular toilet program for fall-susceptible patients and the availability to assistive devices may decrease the risk of fall among such patients.^42^ In sum, the prevention of falls in hospitals may require innovative approaches.

### Patient Medication

The findings of the present study also indicated that medication with sedatives, anticonvulsants, benzodiazepines, ACE inhibitors, anti-infective agents, antihistamine drugs, and chemotherapy drugs increases the rate of falling. A number of studies have marked significant associations among medication, fall incidents, and fall-induced injuries.^43-45^

In this study, antiarrhythmic, vasoactive and ACE inhibitor drugs were classified as a subcategory of cardiac drugs and hypertensive agents, with a greater rate of falling among consumers of ACE inhibitor drugs. In addition, there was no association between the use of antihypertensive agents, which lead to orthostatic hypotension, and the patient falls.^46^ However, intake of hypotonic and diabetic drugs increased the rate of fall incidents among patients. In his study, Schwartz et al^47^ drew up a link between the intake of thiazolidinediones with fall-induced fractures. Also, diabetic patients may have a higher incidence of fall due to hypoglycemia. However, some studies have suggested no impact of diabetes and hypotonic medications on enhancing the risk of falling.^48-50^ In the present study, hypertensive drugs such as diuretics had no effect on patient falls. Shuto et al stated that medical professionals should monitor patients for the initial three days after starting treatment with any of these medications. It is recommended that we modify the use of drugs with a potential fall-increasing nature in terms of dose and time of use. It is also advisable to use drugs under the supervision of a physician.^51^

Consistent with the studies of Mion et al^52^ and Kojima et al,^53^ the role of poly-pharmacy or high dose drug intake on increasing the risk of falls was not confirmed and it seems that intervention studies are required to clarify the causal relationship between poly-pharmacy and fall incidence.

### Fall Circumstances

The results of the present study indicated that the primary activities before or during fall included walking and using the bathroom. Also, some studies suggested evidence of fall incidents for patients who have repeatedly used the bathroom or toilet, which was confirmed by the study of Chelly et al.^6^ The findings have indicated that most falls have occurred when the patient has been alone. Thence, some interventions, including scheduled toileting, regular round of nurses and admission of fall-prone patients in places directly visible from the nursing station or adjacent to the nursing station can be effective.

In terms of fall-induced injuries, more than 63% of fall incidents were associated with minor to severe injuries. Hitcho et al^33^ reported that up to 42% of the falls resulted in injury. Therefore, it is highly advisable to use post-fall analysis tools, environment monitoring and assessment, and interviews with the involved individuals, especially the fallers, to facilitate the development of specific preventive measures in hospitals.

This study had a few limitations: first, it was conducted in a general hospital which may affect its generalization. This problem was somewhat moderated by selecting a large size study population from different specialty wards. The second limitation was that there was a problem of self-reported recall of falls by older patients due to difficulties in speaking in Persian or those who were all alone, which led to underreporting. Finally, considering the limited number of samples in the control group, we could not match the patients for age, gender, etc, which could also lead to biased results.

## Conclusion

In this study, patient-related factors were found to increase the risk of fall. Fall prevention strategies should be linked to the patient characteristics that cause a patient to fall. Therefore, based on our results, strategies should focus on modifiable and effective risk factors such as patients with longer length of stay, history of falls, Morse Falls Scale score, visual acuity, balance, manual transfer aid, urinary incontinence, cancer and diabetes, and taking medications such as chemotherapy, sedatives, anticonvulsants, Benzodiazepines, ACE inhibitors, anti-infective agents, and antihistamine. A focus on post-fall interventions enjoying a patient-oriented approach is required to modify the risk factors associated with fall incidents. The condition and setting where the fall has occurred should be well identified and corrected or at least mitigated. It is suggested that similar studies be conducted focusing on environmental and organizational factors as well as their causality relationships in different types of hospitals.

## Acknowledgements

We thank the patients for their participation and the staff at hospital for their help. Also we would like to thank from Dr. Marzieh Nosrati from faculty of Pharmacy affiliated to Tehran University of Medical Sciences, Tehran, Iran for valuable guidance.

## Ethical issues

This study was conducted with the approval of ethics committee affiliated of Tehran University of Medical Sciences (TUMS), Tehran, Iran.

## Competing interests

Authors declare that they have no competing interests.

## Authors’ contributions

ZN: writing the original article. ZN, ZG: Gathering the data and cooperating in analysing it. MA: project consulter and editing the article. MY: Analysing data. All authors read and approved the final manuscript.

## Authors’ affiliations

^1^Department of Health Care Management, School of Public Health, Ahvaz Jundishapur University of Medical Sciences, Ahvaz, Iran. ^2^Department of Pharmacoeconomics and Pharmaceutical Administration, Faculty of Pharmacy, Tehran University of Medical Sciences, Tehran, Iran. ^3^School of Public Health, Tehran University of Medical Sciences, Tehran, Iran. ^4^Department of Epidemiology and Biostatistics, School of Public Health, Tehran University of Medical Sciences, Tehran, Iran.

## Supplementary files


Supplementary file 1 contains interviewing protocol.

## Key messages

**Implications for policy makers**

- We identify contributory factors affecting fall incidents with the aim of meeting hospital managers’ needs for timely and relevant intervention to improve patient safety in healthcare systems.
- These findings are applicable in all hospitals.

**Implications for public**

The results of the present study reveal factors affecting fall incidents, can be helpful in identifying high risk patients, can encourage the advocacy and the involvement of patients in devising strategies to eliminate such barriers, and can finally help prevent the occurrence of falls, especially in elderly patients.

## References

[1] Hartholt KA, van der Velde N, Looman CW (2010). Trends in fall-related hospital admissions in older persons in the Netherlands. Arch Intern Med.

[2] Haines TP, Hill AM, Hill KD (2013). Cost effectiveness of patient education for the prevention of falls in hospital: economic evaluation from a randomized controlled trial. BMC Med.

[3] Currie LM (2006). Fall and injury prevention. Annu Rev Nurs Res.

[4] Boushon B, Nielsen G, Quigley P, et al. Transforming care at the bedside how-to guide: Reducing patient injuries from falls. Cambridge, MA: Institute for Healthcare Improvement; 2008.

[5] Hitcho EB, Krauss MJ, Birge S (2004). Characteristics and circumstances of falls in a hospital setting: a prospective analysis. J Gen Intern Med.

[6] Chelly JE, Conroy L, Miller G, Elliott MN, Horne JL, Hudson ME (2008). Risk factors and injury associated with falls in elderly hospitalized patients in a community hospital. J Patient Saf.

[7] Healey F, Scobie S, Oliver D, Pryce A, Thomson R, Glampson B (2008). Falls in English and Welsh hospitals: a national observational study based on retrospective analysis of 12 months of patient safety incident reports. Qual Saf Health Care.

[8] Wong CA, Recktenwald AJ, Jones ML, Waterman BM, Bollini ML, Dunagan WC (2011). The cost of serious fall-related injuries at three Midwestern hospitals. Jt Comm J Qual Patient Saf.

[9] Morello RT, Barker AL, Watts JJ (2015). The extra resource burden of in-hospital falls: a cost of falls study. Med J Aust.

[10] Parry SW, Steen N, Galloway SR, Kenny RA, Bond J (2001). Falls and confidence related quality of life outcome measures in an older British cohort. Postgrad Med J.

[11] Stevens JA (2005). Falls among older adults--risk factors and prevention strategies. J Safety Res.

[12] Rubenstein LZ (2006). Falls in older people: epidemiology, risk factors and strategies for prevention. Age Ageing.

[13] Stel VS, Smit JH, Pluijm SM, Lips P (2004). Consequences of falling in older men and women and risk factors for health service use and functional decline. Age Ageing.

[14] Miake-Lye IM, Hempel S, Ganz DA, Shekelle PG (2013). Inpatient fall prevention programs as a patient safety strategy: a systematic review. Ann Intern Med.

[15] Gaebler S (1993). Predicting which patient will fall again and again. J Adv Nurs.

[16] Schwendimann R, Buhler H, De Geest S, Milisen K (2006). Falls and consequent injuries in hospitalized patients: effects of an interdisciplinary falls prevention program. BMC Health Serv Res.

[17] Shorr RI, Chandler AM, Mion LC (2012). Effects of an intervention to increase bed alarm use to prevent falls in hospitalized patients: a cluster randomized trial. Ann Intern Med.

[18] Kwok T, Mok F, Chien WT, Tam E (2006). Does access to bed-chair pressure sensors reduce physical restraint use in the rehabilitative care setting?. J Clin Nurs.

[19] Morse JM, Black C, Oberle K, Donahue P (1989). A prospective study to identify the fall-prone patient. Soc Sci Med.

[20] Baek S, Piao J, Jin Y, Lee SM (2014). Validity of the Morse Fall Scale implemented in an electronic medical record system. J Clin Nurs.

[21] Iinattiniemi S, Jokelainen J, Luukinen H (2009). Falls risk among a very old home-dwelling population. Scand J Prim Health Care.

[22] Anderson C, Dolansky M, Damato EG, Jones KR (2015). Predictors of serious fall injury in hospitalized patients. Clin Nurs Res.

[23] Dunlop DD, Manheim LM, Sohn MW, Liu X, Chang RW (2002). Incidence of functional limitation in older adults: the impact of gender, race, and chronic conditions. Arch Phys Med Rehabil.

[24] Quigley PA, Hahm B, Collazo S (2009). Reducing serious injury from falls in two veterans’ hospital medical-surgical units. J Nurs Care Qual.

[25] Bradley SM, Karani R, McGinn T, Wisnivesky J (2010). Predictors of serious injury among hospitalized patients evaluated for falls. J Hosp Med.

[26] Vassallo M, Vignaraja R, Sharma JC, Briggs R, Allen S (2005). The relationship of falls to injury among hospital in-patients. Int J Clin Pract.

[27] Deandrea S, Bravi F, Turati F, Lucenteforte E, La Vecchia C, Negri E (2013). Risk factors for falls in older people in nursing homes and hospitals A systematic review and meta-analysis. Arch Gerontol Geriatr.

[28] Mayo NE, Korner-Bitensky N, Levy AR (1993). Risk factors for fractures due to falls. Arch Phys Med Rehabil.

[29] Wellens NI, Deschodt M, Boonen S (2011). Validity of the interRAI Acute Care based on test content: a multi-center study. Aging Clin Exp Res.

[30] Healey F, Monro A, Cockram A, Adams V, Heseltine D (2004). Using targeted risk factor reduction to prevent falls in older in-patients: a randomised controlled trial. Age Ageing.

[31] Quigley PA, Hahm B, Collazo S (2009). Reducing serious injury from falls in two veterans’ hospital medical-surgical units. J Nurs Care Qual.

[32] Baloh RW, Enrietto J, Jacobson KM, Lin A (2001). Age-related changes in vestibular function: a longitudinal study. Ann N Y Acad Sci.

[33] Hitcho EB, Krauss MJ, Birge S (2004). Characteristics and circumstances of falls in a hospital setting: a prospective analysis. J Gen Intern Med.

[34] Hauer K, Rost B, Rutschle K (2001). Exercise training for rehabilitation and secondary prevention of falls in geriatric patients with a history of injurious falls. J Am Geriatr Soc.

[35] Capone LJ, Albert NM, Bena JF, Tang AS (2012). Predictors of a fall event in hospitalized patients with cancer. Oncol Nurs Forum.

[36] Spoelstra SL, Given BA, Schutte DL, Sikorskii A, You M, Given CW (2013). Do older adults with cancer fall more often? A comparative analysis of falls in those with and without cancer. Oncol Nurs Forum.

[37] Klein BE, Moss SE, Klein R, Lee KE, Cruickshanks KJ (2003). Associations of visual function with physical outcomes and limitations 5 years later in an older population: the Beaver Dam eye study. Ophthalmology.

[38] Patino CM, McKean-Cowdin R, Azen SP, Allison JC, Choudhury F, Varma R (2010). Central and peripheral visual impairment and the risk of falls and falls with injury. Ophthalmology.

[39] Leat SJ, Zecevic AA, Keeling A, Hileeto D, Labreche T, Brymer C (2018). Prevalence of vision loss among hospital in-patients; a risk factor for falls?. Ophthalmic Physiol Opt.

[40] Krauss MJ, Evanoff B, Hitcho E (2005). A case-control study of patient, medication, and care-related risk factors for inpatient falls. J Gen Intern Med.

[41] Oliver D, Healey F, Haines TP (2010). Preventing falls and fall-related injuries in hospitals. Clin Geriatr Med.

[42] Cumming RG, Sherrington C, Lord SR (2008). Cluster randomised trial of a targeted multifactorial intervention to prevent falls among older people in hospital. BMJ.

[43] Gluck T, Wientjes HJ, Rai GS (1996). An evaluation of risk factors for in-patient falls in acute and rehabilitation elderly care wards. Gerontology.

[44] Salgado R, Lord SR, Packer J, Ehrlich F (1994). Factors associated with falling in elderly hospital patients. Gerontology.

[45] Gales BJ, Menard SM (1995). Relationship between the administration of selected medications and falls in hospitalized elderly patients. Ann Pharmacother.

[46] Verwoert GC, Mattace-Raso FU, Hofman A (2008). Orthostatic hypotension and risk of cardiovascular disease in elderly people: the Rotterdam study. J Am Geriatr Soc.

[47] Schwartz AV, Hillier TA, Sellmeyer DE (2002). Older women with diabetes have a higher risk of falls: a prospective study. Diabetes Care.

[48] Berlie HD, Garwood CL (2010). Diabetes medications related to an increased risk of falls and fall-related morbidity in the elderly. Ann Pharmacother.

[49] Araki A, Ito H (2009). Diabetes mellitus and geriatric syndromes. Geriatr Gerontol Int.

[50] Woolcott JC, Richardson KJ, Wiens MO (2009). Meta-analysis of the impact of 9 medication classes on falls in elderly persons. Arch Intern Med.

[51] Shuto H, Imakyure O, Matsumoto J (2010). Medication use as a risk factor for inpatient falls in an acute care hospital: a case-crossover study. Br J Clin Pharmacol.

[52] Mion LC, Gregor S, Buettner M, Chwirchak D, Lee O, Paras W (1989). Falls in the rehabilitation setting: incidence and characteristics. Rehabil Nurs.

[53] Kojima T, Akishita M, Nakamura T (2011). Association of polypharmacy with fall risk among geriatric outpatients. Geriatr Gerontol Int.

