# Supplementary material for: Risk Factors for Falls in Hospital In-Patients: A Prospective Nested Case Control Study
Source: Int J Health Policy Manag. 2019 Mar 9;8(5):300–6. doi: 10.15171/ijhpm.2019.11 (PMC6571495; doi:10.15171/ijhpm.2019.11)
Supplement: Supplementary file 1 — contains interviewing protocol. [file ijhpm-8-300-s001.pdf]

Supplementary file 1. Interviewing protocol

Following identification of all individuals involved in the fall incident, the necessary information was extracted through filling out a checklist by interviewing the personnel (physician, nurse), patients, and their families.

Questions asked the clinical staff included as below:

- How was that event occurred?
- What were you doing at the time of the fall?
- Was the patient alone at the time of the incident?
- Was the patient examined by a physician after the fall?
- What was ordered for him/her by the physician?
- Did patient get injured*?

In addition, the patients answered some questions, including:

- How did the incident happen?
- Did you ask for help from a nurse before the fall?
- Was necessary training given to you at the time of your hospitalization?
- Did you get injured?
- Were you visited by a physician? What was done for you?
- What is your activity trying to perform at time of fall and reason for activity?

*The patient's condition after the fall was rated by physicians in five levels in checklist: including no injury, minor injury (including patients' complaints of pain, the need for ice packs, bandages, cleaning wounds, swelling, analgesics, etc.), moderate injury (cramps, stretching of the muscles, requiring stitches or bed rest) major injury (fracture, surgery, neurology consultation as the patient's level of consciousness becomes more disturbed) and death.
